# Immune Checkpoint Inhibitors and Targeted Therapies in Early-Stage Non-Small-Cell Lung Cancer: State-of-the-Art and Future Perspectives

**DOI:** 10.3390/cancers17040652

**Published:** 2025-02-14

**Authors:** Lucrezia Barcellini, Simone Nardin, Gianluca Sacco, Michele Ferrante, Giovanni Rossi, Giulia Barletta, Elisa Bennicelli, Chiara Dellepiane, Marco Tagliamento, Beatrice Ramella Pollone, Luca Lucente, Simona Coco, Silvia Marconi, Sara Santamaria, Gian Luca Pariscenti, Carlo Genova

**Affiliations:** 1 Department of Internal Medicine and Medical Specialties (DiMI), School of Medicine, University of Genoa, 16126 Genoa, Italy; lucrezia.barcellini@libero.it (L.B.); gianluca.sacco94@gmail.com (G.S.); m.ferrante9424@gmail.com (M.F.); marco.tagliamento@unige.it (M.T.); beatrice.rampol@gmail.com (B.R.P.); luca_lucente19@yahoo.it (L.L.); 2 U.O. Clinica di Oncologia Medica, IRCCS Ospedale Policlinico San Martino, 16132 Genoa, Italy; giulia.barletta@yahoo.it (G.B.); sara.santamaria@hsanmartino.it (S.S.); 3 U.O. Oncologia Medica 2, IRCCS Ospedale Policlinico San Martino, 16132 Genoa, Italy; giovanni.rossi@hsanmartino.it (G.R.); elisa.bennicelli@hsanmartino.it (E.B.); chiara.dellepiane@hsanmartino.it (C.D.); simona.coco@hsanmartino.it (S.C.); silvia.marconi@hsanmartino.it (S.M.); 4 Department of Thoracic Surgery, IRCCS Ospedale Policlinico San Martino, 16132 Genoa, Italy; gianluca.pariscenti@hsanmartino.it

**Keywords:** non-small-cell lung cancer, personalized medicine, perioperative treatment

## Abstract

**Background**: Approximately 25–30% of non-small-cell lung cancer (NSCLC) patients are diagnosed when the disease is still resectable, although the risk of recurrence is significant. Recently, approaches based on targeted agents or immune checkpoint inhibitors (ICIs) have modified the management of such patients. However, some questions remain unanswered. **Objectives**: Our aim is to assess the current evidence on approaches involving targeted agents and ICIs in resectable NSCLC, to provide an up-to-date overview of the subject, and to identify areas of current debate, **Methods**: We analyzed randomized trials on ICIs and targeted therapies in early-stage NSCLC, published or presented at international oncology meetings throughout the last 5 years. **Results**: Osimertinib and alectinib have shown robust results in the adjuvant setting for molecularly identified patient subgroups, while ICIs have achieved robust data in the neoadjuvant/perioperative setting, with less consistent data on the pure adjuvant approach. Circulating tumor DNA levels may offer a possible biomarker for therapeutic decisions, albeit more prospective data are needed. **Conclusions**: Targeted agents and ICIs are revolutionizing early-stage NSCLC, similarly to what was observed in advanced disease. Prospective studies designed to compare neoadjuvant, adjuvant, and perioperative approaches and to assess the role of circulating biomarkers are warranted.

## Simple Summary

Immunotherapy and targeted therapies for oncogene-addicted disease are leading to a revolution in resectable non-small-cell lung cancer (NSCLC), and several randomized trials are testing and demonstrating their efficacy in the adjuvant, neoadjuvant, and perioperative settings. However, the optimal timing of immunotherapy in these settings is still an open question, while reliable biomarkers for improving patient selection are an unmet need. This review aims to outline new evidence on the use of immune checkpoint inhibitors and targeted therapies in the adjuvant, neoadjuvant, and perioperative settings, highlighting ongoing trials, recent findings, and future perspectives.

## 1. Introduction

Lung cancer is the leading cause of cancer death worldwide, with an estimated almost 2.5 million cases and 1.8 million deaths [1]. Non-small-cell lung cancer (NSCLC) constitutes approximately 85% of all lung cancers, and most patients are diagnosed at locally advanced or metastatic stage, with a 5-year survival rate for all stages of 28% and for metastatic stages of 9%, while only 25% to 30% have resectable disease [2,3]. Despite surgical resection having been considered the gold standard for the treatment of early-stage NSCLC, the recurrence rate is significant in many patients and rises with higher stages [4,5,6]. Several studies have indicated that lobectomy offers the best survival outcomes for patients with resectable tumors, and minimally invasive procedures, such as video-assisted thoracoscopic surgery (VATS) and robotic-assisted thoracic surgery (RATS), may reduce postoperative complications while achieving survival outcomes similar to those of open thoracotomy [7,8,9]. Despite curative surgery, the disease recurs in up to 60% of cases, and early diagnosis and intervention can significantly improve prognosis, with a five-year survival rate of 60–70% for resectable tumors [10,11]. Although neoadjuvant chemotherapy has reported similar outcomes in terms of disease-free survival (DFS) and overall survival (OS) benefit, compared with adjuvant chemotherapy and a higher proportion of patients in the preoperative arm who initiated the planned chemotherapy treatment compared to those in the adjuvant arm (97% vs. 66.2%; *p* < 0.0001), physicians have preferred adjuvant treatment for several years [12,13,14]. Platinum-based combination chemotherapy has remained the therapeutic standard-of-care option in the adjuvant setting for almost twenty years, giving patients an absolute 5-year survival benefit of about 4–5%, according to different meta-analyses [15,16]. Immunotherapy and targeted therapies have led to a revolution in the advanced setting, highlighting the opportunity to exploit possible benefits of these treatment options in earlier stages [17,18]. Neoadjuvant immunotherapy is expected to act against cancer while in site, potentially with improved immune system activation and radiological and pathological demonstration of activity; however, there is a risk of surgery delay or even cancellation, with subsequent lack of curative alternatives [19]. In a recent meta-analysis, neoadjuvant chemo-immunotherapy was associated with a higher incidence of surgical resection and R0 resection than chemotherapy alone, without raising the risk of surgical delays or complications, but increasing the rate of surgery cancellations due to adverse events, especially G ≥ 3 in the chemo-immunotherapy group [20]. There is, to date, no data from phase III trials that directly compare neoadjuvant or perioperative strategies vs. upfront surgery followed by adjuvant options [21].

This review aims to summarize key studies on the efficacy and impact of immune checkpoint inhibitors (ICIs) and targeted therapies in adjuvant, neoadjuvant, and perioperative settings, highlighting ongoing trials, recent findings, and future perspectives.

## 2. Adjuvant Setting

### 2.1. Immune Checkpoint Blockade

The adjuvant treatment aims to improve survival outcomes of resected patients. Historically, adjuvant chemotherapy confers a 5-year OS benefit of only 5% over surgery alone in patients with resected stage IB–IIIA NSCLC. Several phase II and III randomized trials demonstrated the efficacy of using ICIs as an adjuvant treatment after surgery [16,22,23]. Adjuvant immunotherapy has the potential to reverse the immunosuppressive postsurgical microenvironment and to improve patients’ outcomes due to ICIs effectiveness against micrometastatic disease, eliminating minimal residual disease (MRD) [19].

IMpower010 [22] was a phase III randomized trial testing the Programmed Death-Ligand 1 (PD-L1) inhibitor atezolizumab at 1200 mg every 21 days for one year vs. the best supportive care, in patients with resected stage IB to IIIA NSCLC (AJCC Staging Manual, 7th edition) who had received adjuvant platinum doublet-based chemotherapy for 1–4 cycles after surgery. The primary endpoint was DFS, and secondary efficacy endpoints were OS in the intention to treat population (ITT) and DFS in the patients in the stage II–IIIA whose tumors expressed PD-L1 on 50% or more of the tumor cells. At a median follow-up of 32.8 months, atezolizumab showed a benefit in terms of DFS (HR 0.79; 95% confidence interval (CI), 0.64–0.96), and, at a median follow-up of 45.3, the median OS in the ITT population was not estimable (HR 0.995; 95% CI 0.78–1.28). Early results already showed a significant improvement in DFS for patients with PD-L1-positive stage II to IIIA NSCLC (HR 0.66; 95% CI 0.50–0.88) that led to Food and Drug Administration (FDA) approval as an adjuvant treatment following resection and platinum-based chemotherapy for this population. IMpower010 demonstrated a better efficacy for patients with PD-L1 ≥ 50% (95% HR 0.43; 95% CI, 0.27–0.68) than patients with PD-L1 expression < 1% (HR 0.97; 95% CI, 0.71–1.31). The final DFS and second OS interim results after ≥5 years of follow-up were presented, with a minimum follow-up of 60 months, with DFS at 52.0% vs. 46.5% (HR 0.85; 95% CI 0.71–1.01) [24]. In the ITT population, the significance threshold for DFS was not reached (NR), and the OS was similar between arms, although the data were immature. The DFS and OS for the PD-L1 ≥1% and ≥50% stage II-IIIA populations were consistent with previously observed benefits. The patients with an *EGFR* mutation were 10% and 13% in the experimental and control group, and patients with *ALK* rearrangements were 3% and 4%, respectively. The IMpower010 subgroup analysis showed a similar DFS benefit of adjuvant atezolizumab in patients with *EGFR*-positive, *EGFR*-negative, and unknown status. However, it is important to consider the small number of patients with an *EGFR* mutation to interpret these data. Grade (G) ≥ 3 toxicities were 22% and 12% in the experimental arm vs. control arm, respectively. Based on these results, the European Medicines Agency (EMA) approved adjuvant atezolizumab for patients with PD-L1 expression ≥50% and without *EGFR* or *ALK* rearrangements.

KEYNOTE-091/PEARLS [23] was a similarly designed trial, testing the efficacy of pembrolizumab 200 mg every 21 days vs. a placebo in the adjuvant setting for patients with resected stage IB to IIIA NSCLC (AJCC Staging Manual, 7th edition) for up to one year of treatment. Differently from IMpower010, adjuvant platinum doublet chemotherapy was highly recommended but not mandatory, and it was administered to 86% of the enrolled patients. The dual primary endpoints were DFS in the overall population and in the population with PD-L1 ≥ 50%. The secondary endpoints were DFS in the PD-L1 ≥ 1% population, OS in the overall population, and safety in the PD-L1 ≥ 50% population and PD-L1 ≥ 1% population.

At a median follow-up of 35.6 months, pembrolizumab already showed an improvement in DFS in the adjuvant setting (HR 0.76; 95% CI = 0.63–0.91). In the subgroup analysis, patients with PD-L1 < 1% had a stronger DFS benefit (HR 0.78; 95% CI, 0.06–1.03) than those with PD-L1 ≥ 50% (HR 0.82; 95% CI, 0.57–1.18), and patients with PD-L1 between 1 and 49% had the strongest DFS benefit, exceeding that of both the patients with high expressions of PD-L1 and negative PD-L1, observing no difference in survival by PD-L1 status. The data from the final analysis were presented during the 2023 Esmo Immuno-Oncology Congress, showing an improvement in DFS at a median follow-up of 51.7 months (HR 0.81; 95% CI, 0.68–0.96), and consequently, confirming the potential utility of pembrolizumab as an adjuvant therapy after resection for early-stage NSCLC [25]. Grade (G) ≥ 3 toxicities were 34.1% and 25.8% in the experimental arm and control arm, respectively.

Despite that both the IMpower010 and the PEARLS study demonstrated a significant DFS benefit, relevant differences are present between the two trials, including a greater number of patients with stage IIIA disease in IMpower010 (41.1% vs. 28.8%) and a greater proportion of patients with PD-L1 ≥ 1% in PEARLS (60.5% vs. 54.6%). Adjuvant chemotherapy was mandatory only in IMpower010, differently from PEARLS, but most of the patients included in the PEARLS trial received it. The percentage of *EGFR* or *ALK*-driven NSCLC were similar in both studies. Despite a PEARLS subgroup analysis demonstrating no benefit of adjuvant immunotherapy for patients who did not receive adjuvant chemotherapy (HR 1.25; 95% CI, 0.76–2.05), the FDA approved adjuvant pembrolizumab even in this population. Consequently, both atezolizumab and pembrolizumab are approved by the FDA, and the choice of treatment regimen should consider the differences between the studies. Indeed, the relevance of tumor histology was discrepant across studies important, as PEARLS subgroup analyses showed no benefit in patients with squamous NSCLC (HR 1.04; 95% CI, 0.75–1.45), differently than in IMpower010 (HR 0.80; 95% CI, 0.54–1.18); similarly the influence of a tumor’s PD-L1 expression, even if the discrepancy between the trial outcomes was a function of PD-L1 status, remains largely unexplained [26]. In addition, it needs to be also considered that these trials are indicated for patients with stage IB, according to the AJCC Staging Manual, 7th edition, staging. However, tumors measuring 4 cm or larger would be considered at least stage II, according to the AJCC Staging Manual, 8th edition, staging. Consequently, many patients who were eligible in the IMPOWER010 and KEYNOTE-091 trials as stage IIIA, currently under the AJCC Staging Manual, 8th edition, staging would be considered IIIB.

**Figure 1:**
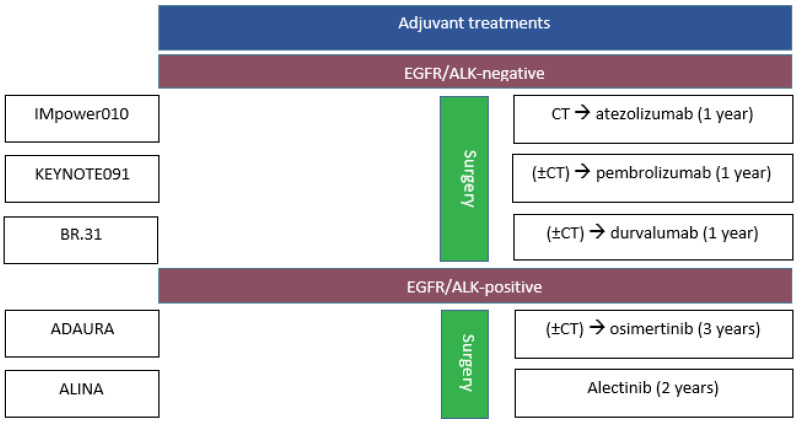
Principal phase II and III adjuvant treatment with ICIs and TKIs in patients with resected NSCLC. CT: chemotherapy.

Finally, BR.31 [27] was a randomized 2:1 phase III trial testing durvalumab every 28 days vs. a placebo for 1 year in 1415 patients with resected stage IB (≥4 cm), II or IIIA NSCLC (AJCC Staging Manual, 7th edition). Patients were stratified by stage, PD-L1 status, adjuvant chemotherapy, treating center, and the extent of nodal sampling, while those with alteration in the *EGFR* or *ALK* gene were excluded. The primary endpoint was DFS in patients with PD-L1 ≥ 25%, whereas the secondary endpoint was DFS in patients with PD-L1 ≥1%. The median follow-up was 60 months, and the median DFS in the *EGFR-/ALK-*population was 60 vs. 54 (HR, 0.89; 95% CI 0.75–10.7), whereas the median DFS was 70 vs. 60 (HR, 0.94; 95% CI, 0.71–1.25) and 60 vs. 60 (HR, 0.99; 95% CI, 0.79–1.25) months for PD-L1 ≥ 25% and PD-L1 ≥ 1%, respectively. Adjuvant durvalumab following R0 resection and optional adjuvant chemotherapy were not associated with significant improvement in DFS outcomes versus (vs.) the placebo, regardless of PD-L1 status. G3–5 toxicities were similar between the arms (27% vs. 21%). The principal phase II and phase III trials of adjuvant treatment with ICIs for resected disease are resumed in Figure 1. The main data from the studies mentioned above are outlined in Table 1.

### 2.2. Targeted Therapies

Nowadays, the landscape of adjuvant therapy for resected NSCLC has undergone dramatic changes in terms of the prescription of new medication, such as the aforementioned immunotherapy, the tyrosine-kinase inhibitors (TKIs), have led to a more targeted treatment of patient resected disease [28,29]. Recent trials guaranteed access to specific TKIs for oncogene-addicted disease, minimizing the risk of relapse. Rearrangement involving the *ALK* gene tends to be less frequent in earlier stages than in metastatic disease as it is reported as less than 5%, likely due to more aggressive clinical behavior with rapidly developing metastases [30,31]. Conversely, the prevalence of an *EGFR* mutation is similar in early and advanced stages, being present in up to 15% of patients [32].

At the present time, molecular analysis in the early stage must be performed for *EGFR* and *ALK*. On the contrary, extended molecular analysis, including oncogenes, such as *ROS1*, *BRAF*, *MET*, *RET*, *NTRK*, and *HER2*, has no direct clinical application because targeted therapies have a limited standard indication for molecular alterations for non-*EGRF* or -*ALK* in the early stage. Consequently, the current NSCLC guidelines in early stage do not recommend an extended molecular panel, even if earlier identification of oncogene alterations could enhance the patients’ selection for perioperative chemo-immunotherapy and clinical trials, as well as improve treatment management for these patients at recurrence [33].

#### 2.2.1. *EGFR* Mutations

In the ADAURA phase III trial [34], 682 patients with completely resected stage IB-IIIA NSCLC who presented common *EGFR* mutations (Ex19del or L858R) were randomized to receive osimertinib 80 mg daily or a placebo for up to 3 years, with the option of adjuvant chemotherapy based on the stage of their disease. After a median follow-up of 44.2 months [35], the median DFS was 65.8 months with osimertinib vs. 21.9 months with the placebo (HR 0.23; 95% CI, 0.18 to 0.30). The overall population (stages IB-IIIA) experienced improved DFS, with a median of 65.8 months compared to 28.1 months (HR 0.27; 95% CI, 0.21 to 0.34). The DFS benefit of osimertinib increased with the disease stage, with an HR of 0.41 for stage IB, 0.34 for stage II, and 0.20 for stage IIIA, respectively. In patients with stage II to IIIA disease, osimertinib showed a benefit in intracranial DFS (HR 0.24; 95% CI, 0.14 to 0.42), with a 3-year probability of intracranial recurrence of 2% with osimertinib vs. 13% with the placebo. In terms of OS, osimertinib provided better outcome compared to the placebo in stage II to IIIA (HR 0.49; 95% CI, 0.33 to 0.73), with a 5-year OS rate of 85% vs. 73% in the overall population (stages IB-IIIA), and the HR for OS was 0.49, with 5-year OS rates of 88% and 78%, respectively. The OS benefit was more pronounced in the higher disease stages, with a 0.44 HR for stage IB, 0.63 for stage II, and 0.37 for stage IIIA, respectively. DFS and OS benefits with osimertinib were observed regardless of adjuvant chemotherapy. Osimertinib confirmed previous results in terms of safety, with 23% of G3 adverse events, especially diarrhea and skin toxicities. Based on the DFS and OS data, osimertinib received authorization by the FDA and EMA as an adjuvant treatment for patients with resected stage IB to IIIA NSCLC and common sensitizing *EGFR* mutations (i.e., deletion of 19 exon or L858R).

#### 2.2.2. *ALK* Rearrangements

In the ALINA phase III trial [36], 257 patients with completely resected *ALK*-translocated stage IB to IIIA NSCLC were randomized to receive oral alectinib 600 mg twice daily for 24 months or adjuvant chemotherapy. In the overall population, the 2-year DFS rate was 93.6% in the alectinib group and 63.7% in the chemotherapy group; the 3-year DFS rate was 88.7% and 54%, respectively. There was a dramatic advantage in terms of risk of recurrence in favor of alectinib (HR 0.24; 95% CI, 0.13 to 0.45), regardless of subgroups by stage, with an HR of 0.21, 0.24, and 0.25, respectively, in stages IB, II, and IIIA. The HR for Intracranial DFS was 0.22 (95% CI, 0.08 to 0.58) in favor of alectinib. No results in terms of OS have been published yet. In terms of safety, G 3–4 toxicities have been reported in 29.7% and 30.8% of patients, respectively. Based on these results, in April 2024, the FDA, and in June 2024, the EMA, approved alectinib as an adjuvant treatment for resected *ALK*-rearranged NSCLC [37,38,39,40,41,42,43,44,45].

Nowadays, advances in diagnostic approaches through gene sequencing are revealing increasingly specific and heterogeneous molecular pathways, and access to next-generation treatments tailored to oncogene targets is expanding over the years. In the advanced setting, there are ten actionable molecular targets with approved targeted therapies: *EGFR* common and uncommon mutations, including *EGFR* exon 20 insertions, *ALK* rearrangements, *ROS1* fusions, *BRAF* V600 mutations, *MET* exon 14 skipping mutations, *HER2* mutation, *KRAS* G12C mutation, and *NTRK* fusions. To each individual molecular pathway, there presents a personalized driven therapy that has been approved based on data from dedicated phase II and phase III randomized trials, in which targeted therapy was compared with chemotherapy in the case of first-generation TKIs, or with earlier-generation TKIs, as seen in the cases of EGFR and ALK. The confirmation of antitumor activity has been translated into benefits in PFS and OS, leading to the approval of targeted therapies in increasingly earlier lines of treatment, up to the first line. Has the approval of Osimertinib and Alectinib in adjuvant settings for resected patients paved the way for the potential future introduction of drugs, such as encorafenib, binimetinib, adagrasib, trastuzumab deruxtecan (T-DXd), or amivantamab, in the postoperative setting? At present, no randomized clinical trials are recruiting in this field. The reasons may be multiple, including difficulties in enrolling patients with resected NSCLC with oncogene addiction, which has a very low incidence, such as HER2, BRAF, or EGFR ex20ins. The most feasible approach in this scenario is the implementation of umbrella trials, designed to guide patients to the most appropriate adjuvant or perioperative treatment based on predictive biomarkers, with a genomic-guided approach. Clinical trials, such as NAUTIKA1, a single-arm phase II study defining perioperative treatment based on biomarkers in resectable stage IB-III NSCLC, hold promise in extending the indications for targeted therapies even to rarer subgroups [46]. Molecular diagnostics and the increasing availability of targeted therapies are opening highly promising horizons for oncogene addiction in NSCLC, not only in the advanced setting but beyond.

The main data from the studies mentioned above are outlined in Table 2. Principal phase II and phase III trials for resected oncogene-driven disease are resumed in Figure 1.

## 3. Neoadjuvant and Perioperative Setting

### 3.1. Chemo-Immunotherapy in the Neoadjuvant Setting

Recent findings highlighted that the efficacy of immunotherapy might be maximized by the presence of the primary tumor with a high neoantigen burden and limited heterogeneity due to the absence of selective pressures associated with treatment; hence, by adding ICIs to chemotherapy in the neoadjuvant setting might lead to an enhancement of neoantigen release, promoting the immune response [19]. On the other hand, adverse events from immunotherapy might delay surgery, increase the risk of progression, and make complete resection more challenging [47].

The CheckMate 816 trial [11] was the first phase III trial of neoadjuvant chemo-immunotherapy in resectable NSCLC to be presented, comparing three cycles of neoadjuvant nivolumab 360 mg every 21 days plus platinum doublet chemotherapy with chemotherapy alone in 358 patients with *EGFR* and *ALK*-negative stage IB (≥4 cm) to IIIA NSCLC (AJCC Staging Manual, 7th edition). The patients were stratified by disease stage, PD-L1 status, and disease histology. At a median follow-up of 29.5 months, the chemo-immunotherapy approach led to improvements in both the co-primary endpoints, pathological complete response (pCR) (24% vs. 2.2%, odds ratio [OR], 13.94) and median event-free survival (EFS) (31.6 vs. 20.8 months, HR 0.63; 97.38% CI 0.43–0.91). In the updated report, with a median follow-up of 41.4 months, the 3-year EFS was 57% vs. 43% in the neoadjuvant nivolumab plus chemotherapy and chemotherapy arms, respectively, with an immature 3-year OS of 78% vs. 64% (HR 0.57; 99.67% CI, 0.30–1.07) [48]. The EFS benefit was greater in patients with higher stage NSCLC than in those with lower stage NSCLC (HR 0.54 for stage IIIA vs. 0.87 for stage IB and II), patients with PD-L1 expression ≥ 50% (HR 0.24) than in patients with tumor PD-L1 of 1% to 49% (HR 0.58) or PD-L1 lower than 1% (HR 0.85), and in patients with non-squamous NSCLC than squamous one (HR 05 vs. HR 0.77, respectively). Data from the 4-year update of the phase III CheckMate 816 study were presented, and the median EFS with nivolumab plus chemotherapy was 43.8 months (95% CI, 30.6-NR) vs. 18.4 months (95% CI, 14.0–26.7) with chemotherapy alone (HR 0.66; 95% CI, 0.49–0.90) [49]. The median OS was NR in both arms with a hazard ratio of 0.71 (98.36% CI, 0.47–1.07; unstratified HR, 0.69; 95% CI, 0.49–0.97). Notably, the addition of ICIs improved quality of life scores and did not increase the incidence of Grade 3–4 adverse events (33.5% vs. 36.9% with chemotherapy). Neutropenia was the main toxicity (8.5% and 11.1% in the experimental and control arm, respectively). In summary, the CheckMate 816 trial led to a paradigm shift for resectable early-stage NSCLC without negatively affecting surgical feasibility, leading to the approval by the FDA in March 2022 and the EMA in June 2023 of nivolumab + chemotherapy for neoadjuvant treatment for stage IB to III NSCLC patients.

TD-FOREKNOW [50] was a randomized phase II trial assessing the efficacy and safety of neoadjuvant camrelizumab plus chemotherapy vs. chemotherapy alone for patients with resectable stage IIIA or IIIB NSCLC. Patients were randomly assigned to receive three cycles of camrelizumab 200 mg every 21 days plus chemotherapy (nab-paclitaxel and platinum) or chemotherapy alone, followed by surgery after 4 to 6 weeks. The primary endpoint was pCR, and the secondary endpoints were major pathologic response (MPR), Objective Response Rate (ORR), EFS, and safety. At a median follow-up of 14.1 months, the pCR rate was 32.6% (95% CI, 19.1–48.5%) with camrelizumab plus chemotherapy vs. 8.9% (95% CI, 2.5–21.2%) with chemotherapy alone (ORR 4.95; 95% CI, 1.35–22.37). The MPR rates were 65.1% (95% CI, 49.1–79.0%) with camrelizumab plus chemotherapy and 15.6% (95% CI, 6.5–29.5%) with chemotherapy alone. MPR was higher in patients receiving camrelizumab plus chemotherapy either for stage IIIA (30% vs. 8.3%) or stage IIIB patients (38.5% vs. 11.1%). The ORRs were 72.1% (95% CI, 56.3–84.7%) with camrelizumab plus chemotherapy and 53.3% (95% CI, 37.9–68.3%) with chemotherapy alone. The median EFS and DFS were not reached in either group, with an HR of 0.52 (95% CI, 0.21–1.29) and 0.54 (95% CI, 0.19–1.54), respectively. Grade ≥3 toxicities were reported in 25.6% of patients in the experimental arm vs. 11.1% in the control arm. The most common neoadjuvant treatment-related adverse event of G ≥ 3 was neutropenia, with an incidence of 7.0% vs. 11.1%, respectively. No treatment-related deaths were reported.

The NeoCOAST [51] was a phase II randomized trial investigating neoadjuvant immunotherapy combined with novel, experimental agents for the treatment of patients with stage IA3–IIIA resectable NSCLC (AJCC, 8th edition).

Eighty-three patients underwent a single treatment cycle, given over a 4-week period, prior to the scheduled surgery. A total of 26 patients received durvalumab alone, 21 received durvalumab combined with oleclumab (anti-CD73), 20 received durvalumab with monalizumab (anti-NKG2A), and 16 received durvalumab in combination with danvatirsen (anti-STAT3 antisense oligonucleotide). The baseline disease characteristics were consistent with the target patient population and well-balanced across the four treatment arms. However, the durvalumab + monalizumab arm had a higher percentage of patients with stage IA3 disease at baseline (30.0%), while the durvalumab monotherapy arm had a lower percentage of patients with stage IIIA disease (7.4%)

The primary endpoints were pCR and MPR, reported as follows: 3.7% (95% CI, 0.1–19.0) and 11.1% (CI 95%, 2.4–29.2), respectively, in the durvalumab monotherapy arm, 9.5% (95% CI, 1.2–30.4) and 19.0% (95% CI, 5.4–41.9), respectively, in the durvalumab + oleclumab arm, 10.0% (95% CI, 1.2–31.7) and 30.0% (95% CI, 11.9–54.3), respectively, in the durvalumab + monalizumab arm, and 12.5% (95% CI, 1.6–38.3) and 31.3% (95% CI, 11.0–58.7), respectively, in the durvalumab + danvatirsen arm. The higher MPR rates in the durvalumab + oleclumab and durvalumab + monalizumab groups were associated with a more pronounced immunogenic profile, according to the multiplatform immune profiling. In total, 95.2% of patients completed the scheduled neoadjuvant therapy, in particular, 96.3% of patients in durvalumab monotherapy group, 85.7% in the durvalumab + oleclumab group, 95.0% in the durvalumab + monalizumab group, and 93.8% in the durvalumab + danvatirsen group. As far as the safety profiles are concerned, G ≥ 3 toxicities were reported in 4.8% of the patients in the durvalumab + oleclumab arm, 0% in the durvalumab + monalizumab arm, and 6.3% in the durvalumab + danvatirsen arm vs. 0% in the durvalumab monotherapy arm.

Finally, NEOSTAR [52] was a phase II randomized trial testing neoadjuvant therapy with three cycles of nivolumab + ipilimumab vs. nivolumab, followed by surgery in 44 patients with previously untreated stage I to IIIA NSCLC, being the first trial to combine PD-1 and CTLA-4 blockade in this setting [26]. The primary endpoint was the MPR rate, achieved by 38% of patients in the nivolumab + ipilimumab arm and 22% in the nivolumab arm. Compared with nivolumab, nivolumab + ipilimumab resulted in a higher pCR rate (10% vs. 38%) and less viable tumor (50% vs. 9%). When chemotherapy was added [53], the MPR rates increased to 32.1% (7/22, CI 80%, 18.7–43.1%) in the nivolumab + chemotherapy arm and 50% (11/22, CI 80% 34.6–61.1%) in the ipilimumab + nivolumab + chemotherapy arm, and the primary endpoint was met in both arms. Furthermore, it has been noticed that the percent of residual viable tumor (%RVT) was only 4.5% in the chemotherapy arm, compared to 50% in the nivolumab + chemotherapy arm. Adverse events in NEOSTAR were similar to those in other trials in this setting, and the rate of G ≥ 3 complications were comparable across the different treatment arms [26,53,54]. However, patients treated with both ipilimumab and nivolumab experienced more surgical complications than those treated with nivolumab + chemotherapy (65% vs. 32%).

**Figure 2:**
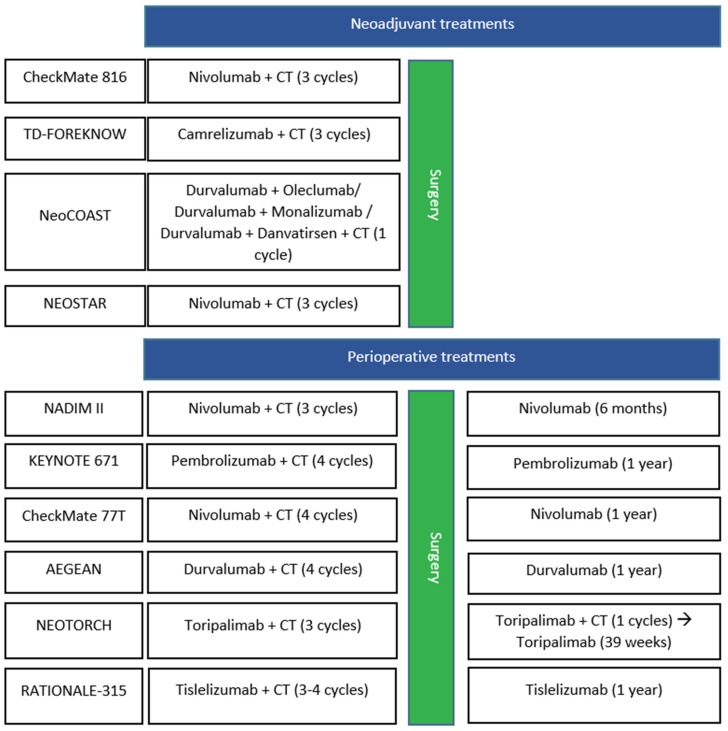
Principal phase II and III neoadjuvant and perioperative treatment with ICIs in patients with resected NSCLC. CT: chemotherapy.

Principal phase II and phase III trials of neoadjuvant treatment with ICIs for resected disease are resumed in Figure 2. The main data from the studies mentioned above are outlined in Table 3.

### 3.2. Perioperative Chemo-Immunotherapy

Adjuvant treatment with ICIs demonstrated their ability to reduce the risk of relapse, while a combination of chemotherapy and immunotherapy has been used in neoadjuvant settings to improve their efficacy, based on the biological rationale mentioned before. This approach has now been incorporated into the NCCN Clinical Practice Guidelines in Oncology (NCCN Guidelines) for the management of early-stage NSCLC [55]. Many ongoing trials are currently investigating the use of ICIs in the perioperative setting, either as monotherapy or combined with chemotherapy [47]. Recent studies have investigated perioperative treatment strategies, which combine the benefit of preoperative and postoperative administration of ICIs to maximize both tumor shrinkage and systemic control before surgery and eliminate potential micrometastatic disease [19]. Based on the encouraging results of the NADIM trial [56], which evaluated neoadjuvant treatment with nivolumab plus paclitaxel–carboplatin, followed by resection and 1 year of adjuvant nivolumab, the randomized phase II NADIM II trial was launched. The NADIM II trial [57] focused on refining multimodality therapy for locally advanced NSCLC, adding nivolumab 360 mg every 21 days to neoadjuvant chemotherapy and, after surgery, nivolumab 480 mg once every 28 days for 6 months in patients with stage IIIA-IIIB (up to N2 only) NSCLC (AJCC Staging Manual, 8th edition). The evaluation of *EGFR* and *ALK* rearrangements was mandatory, and patients with an oncogene-addicted NSCLC were not admitted. This trial demonstrated that it was feasible to perform definitive resections after chemo-immunotherapy, and in fact, it was significantly more likely to be achieved compared to chemotherapy alone (93% vs. 69%; OR 5.96; *p* = 0.008). The NADIM II randomized 86 patients, and at a median follow-up of 26.1 months, the pCR rate was higher in the nivolumab + chemotherapy group (36.8% vs. 6.9%, *p* = 0.0068), and a similar result was reached for the MPR (52.6% vs. 13.8%). The nivolumab + chemotherapy group had significantly better EFS (HR, 0.47; 95% CI 0.25–0.88) and 2-year OS (85.0% vs. 63.6%; HR 0.43, 95% CI, 0.19–0.98). An improved OS in patients who completed the adjuvant treatment compared to patients with an incomplete adjuvant treatment was demonstrated by a post hoc analysis, with an HR of 0.29 (95% CI, 0.05–1.76 vs. 3.52; 95% CI, 0.93–3.24) [57]. In addition, this trial demonstrated, for the first time, that a neoadjuvant chemo-immunotherapy can significantly increase not only DFS (NR vs. 15.4 months with chemotherapy; HR 0.47, 95% CI 0.25–0.88) but also OS (median OS NR in both the arms; HR 0.43, 95% CI 0.19–0.98). Toxicities were reported in 19% of patients in perioperative treatment, including neutropenia (7%), diarrhea (4%), and asthenia (4%).

The KEYNOTE-671 trial [58] was the first phase III trial testing immunotherapy in a perioperative setting. Neoadjuvant platinum doublet chemotherapy with either pembrolizumab 200 mg every 21 days or a placebo for four cycles was administered to 797 patients with stage II–IIIB NSCLC, per AJCC Staging Manual, 8th edition, criteria, followed by surgery, and then an additional year of pembrolizumab 200 mg every 21 days or a placebo for a maximum of 13 cycles. Patients with an *EGFR* mutation or *ALK* rearrangements were admitted and represented 6.5% and 7.0% of the population in the study, respectively. However, molecular testing was not mandatory in this trial, and consequently, very few patients with EGFR mutations or ALK rearrangements were identified, limiting any insights in these subgroups. In addition, participants with identified *ALK* translocation were excluded from the analysis due to the low number (21 participants), while the *EGFR* mutated population benefited from neoadjuvant chemo-immunotherapy in terms of EFS (HR 0.09; 95% CI, 0.01–0.74). After a median follow-up of 25.2 months, there was a significant improvement in EFS with the addition of pembrolizumab (47.2% vs. 18.3%; 2-year EFS, 62.4% vs. 40.6%; HR 0.58; *p* < 0.001). Adding pembrolizumab to chemotherapy was also associated with a significant improvement in pCR (18.1% vs. 4.0%). The MPR rate was 30.2% in the experimental arm compared with 11.0% in the control arm. Notably, even patients who received adjuvant pembrolizumab and did not achieve MPR showed an improvement in EFS, compared to the placebo. This suggests that perioperative ICIs treatment may also offer a clinically meaningful EFS benefit for such patients [58]. Pembrolizumab was associated with improved EFS both in patients who achieved a pCR (HR 0.33; 95% CI, 0.09–1.22) and those who did not (HR 0.69; 95% CI, 0.55–0.85). The OS benefit was superior with pembrolizumab (NR vs. 52.4 months with the placebo; HR 0.72, 95% CI 0.54–0.99), regardless of histology, PD-L1 expression, or stage of disease [59]. The 3-year OS was significantly longer in the pembrolizumab group (71% vs. 64%; HR, 0.72; 95% CI, 0.56–0.93). In particular, 43% of patients had squamous histology (HR, 0.57 vs. 0.58 of non-squamous histology), one-third had stage II disease (HR, 0.65 vs. 0.54 of stage III disease), 45% had cN2 disease, and one-third of tumors had PD-L1 expression of ≥50% (HR, 0.42 vs. 0.51 vs. 0.77 of PD-L1 1–49% and <1%, respectively). At the second interim analysis, with a median follow-up of 36.6 months, the median EFS was 47.2 months (95% CI, 32.9-NR) in the experimental group and 18.3 months (95% CI, 14.8–22.1) in the placebo arm (HR 0.59; 95% CI 0.48–0.72), whereas the OS rates were 71% (95% CI, 66–76) and 64% (95% CI, 58–69), respectively (HR 0.72; 95% CI 0.56–0.93) [60]. A total of 45% vs. 38% of patients presented drug-related G ≥ 3 toxicities, respectively. In particular, the most common were hematological, including anemia (7.3% vs. 5.5%) and neutropenia (20.7% vs. 19.5%). Importantly, being the first perioperative immunotherapy trial showing an OS benefit in early-stage NSCLC, the FDA and EMA approved perioperative pembrolizumab in October 2023 and in March 2024, respectively, in patients with stage II-III NSCLC [26].

The CheckMate 77T [61] is a double-blind phase III trial that randomized 452 patients with stage II to IIIB (N2) NSCLC to receive neoadjuvant nivolumab 360 mg every 21 days + chemotherapy for four cycles and adjuvant nivolumab 480 mg every 28 days up to 1 year or a neoadjuvant chemotherapy + perioperative placebo. Evaluation of *EGFR* and *ALK* rearrangements was mandatory, and patients whose tumor harbored any of such alterations were not admitted. At the first interim analysis, with a median follow-up of 25.4 months, nivolumab resulted in significantly longer EFS than chemotherapy (1.5-year EFS 70.2% vs. 50%, median EFS NR vs. 18.4 months; HR, 0.59; 97.4% CI, 0.42–0.81). An MPR occurred in more patients in the nivolumab group than in the chemotherapy group (35.4% vs. 12.1%; OR, 4.01; 95% CI, 2.48–6.49), as did the pCR (25.3% vs. 4.7%; OR, 6.64; 95% CI, 3.40 to 12.97). The OS was NR in both arms. The EFS benefit was reported in the subgroup analysis: the benefit was higher in stage IIIA (HR 0.51) than stage II (HR 0.81), and in N2 disease (HR 0.46) than in N1 (HR 0.58). A major benefit was also reported in patients with tumor PD-L1 expression of 1% or more (HR 0.52; 95% CI, 0.35 to 0.78), compared to those with a PD-L1 negative tumor (HR 0.73; 95% CI, 0.47 to 1.15). Among treated patients, G ≥ 3 treatment-related adverse events (TrAEs) occurred in 32.5% and 25.2%, respectively, leading to treatment discontinuation in 11.0% and 4.8% of patients, respectively. The most common G ≥ 3 TrAEs in the nivolumab and chemotherapy groups was a decreased neutrophil count (10.1% and 6.5%, respectively). The FDA and EMA approved perioperative nivolumab in October 2023 and in January 2024, respectively.

The global AEGEAN trial [62] was a phase III randomized trial, enrolling 802 patients with stage IIA-IIIB (cN2, mediastinal lymph node involvement) NSCLC, by AJCC Staging Manual, 8th edition, criteria and randomized to receive either 4 cycles of neoadjuvant chemotherapy plus durvalumab 1500 mg every 21 days, followed by 12 cycles of adjuvant durvalumab 1500 mg every 4 weeks, or neoadjuvant chemotherapy followed by an adjuvant placebo. The patients scheduled for pneumonectomy or with T4 disease (for any criterion other than tumor size, diameter ≥ 7 cm) were excluded from enrolment in the trial. Any PD-L1 expression status was allowed, and up to one-third of tumors had PD-L1 expression ≥ 50%, and almost half of the patients in both arms had N2 disease. Data from 62 patients with documented *EGFR* or *ALK* rearrangements were excluded from the efficacy analyses in the modified intention-to-treat population, but primary analyses showed an HR for disease progression of 0.86 in the EGFR mutated subgroup. According to the primary endpoints, at a median follow-up of 11.7 months, the pCR rate was higher in patients in the experimental arm (17.2% vs. 4.3%; *p* = 0.004), with an MPR of 33.3% vs. 12.3%. As can be expected, the MPR rate was higher in the N2 single patients (37.6% vs. 16.7%) than those with N2 multiple stations (11.8% vs. 10.0%), and in patients with PD-L1 > 50% (50.5% vs. 12.1%; HR, 0.60) than other subgroups (HR, 0.70 and 0.76, respectively, in patients with PD-L1 expression 1–49% and <1%). The EFS was NR vs. 25.9, while the 2-year EFS was 63.3% vs. 52.4% (EFS HR, 0.68; 95% CI, 0.53–0.88). Durvalumab was associated with a similar response rate in non-squamous and squamous NSCLC (HR 0.69 vs. 0.71). The benefit in EFS was observed regardless of disease stage, even if it was higher in stage IIIA patients (HR 0.57) than in stage II patients (HR 0.76) or stage IIIB patients (HR 0.83). Toxicities were similar and reported in 42.4% vs. 43.2%, respectively, in the two arms, in particular, anemia (6.5% vs. 6.5%), neutropenia (9.0% vs. 9.5%), and pneumonitis (1.2% vs. 1.0%), while other most common toxicities were reported in <1% of cases. The FDA and EMA approved perioperative durvalumab in August 2024.

The NEOTORCH study [63] was a randomized phase III trial that randomly assigned 404 patients with stage II-III NSCLC, by AJCC Staging Manual, 8th edition, criteria to receive neoadjuvant chemotherapy combined with toripalimab 240 mg every 21 days or chemotherapy with a placebo, both for 3 cycles before surgery and 1 cycle after the procedure, followed by single-agent toripalimab 240 mg every 21 days or a placebo monotherapy for 13 cycles. The study population was characterized by 77% of patients with squamous histology, 70% with N2 disease, and 65% with PD-L1 expression ≥ 1%. The evaluation of *EGFR* mutations and *ALK* rearrangements was mandatory, and positivity resulted in ineligibility for trial participation. At a median follow-up of 18.3 months, both primary endpoints of the MPR rate and EFS were met. The MPR rate was 48.5% for the toripalimab group vs. 8.4% for the chemotherapy-alone group (*p* = 0.0001); corresponding pCR rates were 24.8% and 1.0%. The median EFS was NR vs. 15.1 months (HR 0.40; 95% CI, 0.28–0.57), while the 2-year EFS was 64.7 vs. 38.7%. The OS outcomes were also more favorable with toripalimab (NR vs. 30.4 months; HR 0.62, 95% CI 0.38–0.99). The 2-year OS was 81.2% vs. 74.3%, respectively, in the two arms. The results reported were similar both in the different PD-L1 expression groups, PD-L1 < 1% (HR 0.65; 95% CI 0.33–1.23), 1–49% (HR 0.31; 95% CI 0.17–0.54), and ≥50% (HR 0.31; 95% CI 0.15–0.60), and tumor histology, non-squamous disease (HR 0.54; 95% CI 0.27–1.09), and squamous disease (HR 0.35; 95% CI 0.24–0.53). The EFS benefit was reached both in stage IIIA (HR 0.44) and stage IIIB (HR 0.30) disease. Drug-related toxicities involved 63.4% vs. 54.0% of patients, respectively. In particular, the most common were anemia (8.9% vs. 7.9%) and neutropenia (33.7% vs. 29.7%).

RATIONALE-315 [64] was a randomized phase III trial, testing the use of tislelizumab vs. a placebo in combination with perioperative chemotherapy. Patients with untreated stage II-IIIA squamous or non-squamous NSCLC were enrolled and randomly assigned to receive neoadjuvant platinum-based doublet chemotherapy plus tislelizumab 200 mg or the placebo every 3 weeks, followed by surgery and adjuvant tislelizumab 400 mg or the placebo every 6 weeks, respectively. The co-primary endpoints were EFS and MPR. At a median follow-up of 22.0 months, tislelizumab demonstrated a statistically significant improvement in EFS compared to the placebo (HR 0.56; 95% CI 0.40–0.79). The MPR rate was notably higher in the tislelizumab group (56%; 95% CI 50–63) compared to the placebo group (15%; 95% CI 11–20). Additionally, the pCR rate was significantly greater in the tislelizumab group than in the placebo group (41% vs. 6%; *p* < 0.0001). A trend toward improved OS was observed in favor of the tislelizumab group over the placebo group, with a stratified HR of 0.62 (95% CI 0.39–0.98). However, this trend did not reach statistical significance at the interim analysis (*p* = 0.019). The median OS was not reached in either treatment group. The 24-month OS rate was 89% (95% CI 83–92) for the tislelizumab group and 79% (95% CI 73–85) for the placebo group. Grade 3 or worse adverse events occurred in 72% of patients in the tislelizumab group and 66% of patients in the placebo group. The most frequent Grade 3 or worse treatment-related adverse event was a decreased neutrophil count, occurring in 61% of the tislelizumab group and 59% of the placebo group.

The main data from the studies mentioned above are outlined in Table 3. The most relevant phase II and phase III trials of perioperative treatment with ICIs for resected disease are reported in Figure 2.

### 3.3. Future Perspectives in the Neoadjuvant and Perioperative Setting

As stated before, targeted therapies are a cornerstone for advanced NSCLC and are carving out important space in the adjuvant treatment, especially for *EGFR* and *ALK*-positive tumors. However, in the neoadjuvant setting, few data are available. The phase III NeoADAURA trial is evaluating the use of osimertinib with or without chemotherapy, compared to chemotherapy alone, for resectable stage II-IIIB N2 NSCLC with an *EGFR* mutation [59]. The chemotherapy compound is represented by platinum agents plus pemetrexed for three cycles, and the primary endpoint is the major pathological response. Even if no results have been published yet, previous phase II trials reported positive results with this approach [65,66]. Regarding *ALK*-positive NSCLC, the most relevant phase II trials evaluating the use of alectinib as neoadjuvant or perioperative therapy are NAUTIKA1 and ALNEO [46,67]. Preliminary efficacy data from NAUTIKA1, evaluating alectinib in stage IB-IIIB squamous or non-squamous NSCLC, reported a major pathological response and complete response in 71.4% and 28.6% of patients, respectively, even if only 7 patients were included in the analysis [46]. The ALNEO efficacy data were presented at the 2024 World Conference on Lung Cancer (WCLC 2024): the MPR was reported in 39% of cases and pCR in 17% out of 18 patients evaluated. Other targeted therapies for *MET*, *RET*, and *KRAS* (G12C) driver mutations are ongoing but yet in phase II trials [67]. Novel immunotherapy combinations, shifting from advanced settings, have been evaluated in the early stage. T-cell immunoglobulin and immunoreceptor tyrosine-based inhibitory motif domains (TIGITs) are novel targets for immunomodulatory drugs (anti-TIGITs). One of these, tiragolumab, has been evaluated as a first-line treatment in combination with atezolizumab in PD-L1 selected NSCLC [68]. A phase II trial is evaluating the addition of tiragolumab to atezolizumab as a neoadjuvant and adjuvant treatment, with or without chemotherapy, for resectable stage II, IIIA, or IIIB NSCLC [69]. Another immunomodulatory drug has lymphocyte-activation gene 3 (LAG-3) as the target, relatlimab. In a randomized phase II trial, relatlimab is being evaluated in association with nivolumab as a neoadjuvant treatment for IB, II, or IIIA NSCLC [70]. Other combinations, including immune checkpoint blockage with CD137, IDO1, and OX40 inhibitors, are going to be evaluated in advanced or unresectable settings only, but their potential results could move them to an early setting in a few years [71]. Notably, antibody-drug conjugates are enriching the treatment landscape for all malignancies, and clinical trials involving such drugs in resectable NSCLC are being conducted, as well. In patients with NSCLC, results from phase III studies in advanced settings have been already published [72,73,74,75,76]. In neoadjuvant therapy, the phase 2 NeoCOAST-2 trial had a treatment arm with datopotamab-deruxtecan (Dato-DXd), durvalumab, and single-agent platinum chemotherapy. Recent data were presented during the WLCL 2024: treatment with Dato-DXd plus durvalumab plus single agent platinum chemotherapy obtained a pathological complete response in 34.1% of patients, compared to 26.7% and 20% of treatments with monalizumab plus durvalumab plus platinum doublet chemotherapy and oleclumab plus durvalumab plus platinum doubled chemotherapy, respectively [77]. Another phase II trial is recruiting patients with stage II-III NSCLC to receive four cycles of neoadjuvant pembrolizumab plus sacituzumab govitecan, followed by surgery, and 1 year of adjuvant pembrolizumab (NCT06055465). This study is still ongoing without efficacy data published yet.

In a mouse model of hepatocellular carcinoma, vascular normalization induced by tyrosin-kinase inhibitors of the vascular endothelial growth factor receptor-2 (VEGFR2) antibody can enhance the effectiveness of ICIs by reshaping the immune microenvironment [78]. Indeed, a first-line treatment for hepatocellular carcinoma is related to the combination of atezolizumab plus bevacizumab [79]. Qiao et al. evaluated this approach in a humanized mouse model of NSCLC, combining pembrolizumab plus bevacizumab [80]. The results indicated that combination therapy could suppress tumor growth by converting a tumor with low immunoreactivity into a “hot” tumor, as evidenced by increased infiltration of CD8+ granzyme B+ cytotoxic T cells. In a phase II trial, Zhao et al. evaluated the combination of camrelizumab, an anti-PD-1, plus apatinib, an anti-VEGFR-2, as a neoadjuvant treatment for resectable NSCLC. Among 78 patients treated, 65 underwent surgery, 57% (IC 95%, 44–69%) had an MPR, and among them, 23% achieved an MPR (95% CI, 14–35%) [81]. Regarding safety, only 5% of patients suffered from Grade 3 toxicities, and the authors reported no Grade 4 or 5 treatment-related adverse events. Similarly, Aokage et al. conducted a phase II trial on neoadjuvant treatment with pembrolizumab plus ramucirumab, a VEGFR-2 antibody antagonist, for PD-L1 positive NSCLC, stage IB-IIIA [82]. The 24 enrolled patients obtained an MPR in 50% of cases (95% CI, 31.9–68.1%), and 6 patients achieved a pCR. Grade 4 and 5 adverse events did not occur, and Grade 3 events were reported in 37.5% of patients. A phase II, two-arm study evaluated the use of camrelizumab plus apatinib in stage II-III unresectable NSCLC. Among the 21 patients who received two to four cycles of neoadjuvant treatment with camrelizumab plus apatinib, 9 became eligible for surgery, achieving 55.6% (95% CI, 21.2–86.3%) of an MPR, including 1 with a pCR [83].

Lastly, cancer vaccines could represent a novel approach in a curative setting. They could be cellular, viral vector, or molecular (e.g., peptide, DNA, or RNA) [84]. The rationale is to use the tumor-associated antigen or tumor-specific antigen to create a vaccine that could enhance patients’ immune systems [85]. Many phase I or II trials have been conducted, but for the early stage, the administration of cancer vaccines followed surgical removal as an adjuvant treatment only, and their role as neoadjuvant administration is still to be stated [86,87]. Future lung cancer management will require comprehensive, patient-centered care, involving the collaboration of various stakeholders in the treatment of early-stage disease [88].

## 4. Timing of Immunotherapy: A Comparison Between Neoadjuvant and Adjuvant Treatment and Between Neoadjuvant and Perioperative Chemo-Immunotherapy

Despite the feasibility of neoadjuvant chemo-immunotherapy, which has been confirmed in the previously described randomized phase II-III clinical trials, the question of the optimal timing of immunotherapy for resectable NSCLC is still unanswered [57,58,61,62,63]. A recent analysis using retrospective data from the National Cancer Database, which focused on patients with stage II-IIIB resected NSCLC who received neoadjuvant or adjuvant chemo-immunotherapy between 2015 and 2020, showed that neoadjuvant chemo-immunotherapy provides a significant overall survival benefit compared to adjuvant chemo-immunotherapy for these patients [89]. It is also worth questioning if the incremental benefit of an additional year of immunotherapy after neoadjuvant chemo-immunotherapy and whether an entire year of adjuvant immunotherapy is necessary to achieve clinical benefit.

Data support the theory that immunotherapy given in the neoadjuvant phase could elicit a more enhanced systemic antitumor response when neoantigen-bearing tumor and antitumor T cells are not yet surgically resected, allowing a greater antitumor response to be exerted by effector memory T cells [26,90,91,92]. For this reason, the presence of a whole tumor could allow activation of a broader and more diverse immune response, leading to an early induction of immune response and the potential to eradicate micrometastases [19]. Furthermore, a rapid control of micrometastases present at the moment of diagnosis may lead to a long-term benefit in terms of outcome [93]. The aforementioned trials demonstrated better adherence rates among patients who received a neoadjuvant treatment (85% to 94% in CheckMate 816 and AEGEAN trials) compared to patients in the adjuvant trials (52% to 64% in IMpower010 and PEARLS) [11,22,23,62]. This result might be linked to the patient status, as indeed, participants from adjuvant therapies may feel less well after surgery or chemotherapy, resulting in lower medication compliance or motivation to continue systemic treatment [94]. In addition, in both the CheckMate 816 and KEYNOTE-671 trials, fewer pneumonectomies were performed in the chemo-immunotherapy arm compared with the chemotherapy alone group (13.9% and 19.0% vs. 11.4% and 12.3%, respectively), underscoring the potential for tumor downstaging with neoadjuvant treatment, which may enable higher rates of minimally invasive surgery [11,58]. On the other hand, the possible risk of losing the curative window due to the delay or cancellation of potentially curative surgery caused by drug toxicity or disease progression if patients do not respond to surgery has to be considered [94]. In addition, there is also the need to counterbalance the toxicity with potential immune-related adverse events, the high cost of this additional year of visits and labs, and infusions for the patient. A recent meta-analysis indirectly demonstrated that the addition of ICIs in the adjuvant phase was not associated with a significant improvement in EFS or OS for patients with resectable NSCLC who received neoadjuvant combined treatment with ICIs and chemotherapy [66]. By contrast, during the WCLC 2024, a patient-level data analysis of Checkmate 816 and Checkmate 77T was presented [95]. In this analysis, perioperative nivolumab gave a notable improvement over neoadjuvant nivolumab + chemotherapy in terms of EFS (HR 0.61, CI 95% 0.39–0.97), which was superior when pCR is reached (HR 0.58, CI 95%, 0.14–2.40) compared to cases where pCR was not reached (HR 0.65, CI 95% 0.40–1.06). Similarly, the benefit of the perioperative approach was superior in cases where PD-L1 expression was <1% (HR 0.51, CI 95%, 0.28–0.93) compared to cases where PD-L1 was ≥1% (HR 0.86, CI 95%, 0.44–1.70). Notably, while the observations of this patient-level analysis support perioperative chemo-immunotherapy over neoadjuvant chemo-immunotherapy, some biases have been reported as the analysis only included patients who underwent surgical resection in Checkmate 816 and, for CheckMate 77T, only patients receiving at least one administration of adjuvant nivolumab were included, thus limiting the interpretation of the results. Hence, randomized trials prospectively evaluating neoadjuvant vs. adjuvant vs. perioperative immunotherapy are awaited. In the CheckMate 816 trial, patients achieving tumor pCR experienced a 2-year EFS rate exceeding 90%, contrasting with approximately 50% among those without pCR (HR, 0.13; 95% CI, 0.05–0.37). This finding provides an attractive basis to investigate whether the extent of pathologic response observed in surgery could represent a biomarker guiding adjuvant treatment escalation or de-escalation strategies [92]. In the meantime, patients’ management regarding the timing and duration of immunotherapy will remain individualized [26]. Decisions about whether to pursue adjuvant therapy after neoadjuvant chemo-immunotherapy remain determined by clinical judgment and shared decision-making with the patient about their priorities, patient molecular biomarkers, tolerability to prior treatment, preferences regarding care goals, and susceptibility to autoimmune toxic effects. High-dimensional multiomics investigations within the neoadjuvant treatment framework would be necessary [92,96].

## 5. Patient Selection for Immunotherapy: Role of ctDNA

Circulating tumor DNA (ctDNA) is being increasingly used in clinical practice as an early detection biomarker of recurrence, showing its utility in detecting molecular residual disease and in monitoring response to treatment to optimize treatment and surveillance strategies [97]. The ctDNA is an emerging innovation that has been demonstrated to predict outcomes in patients receiving ICIs, including both those with locally advanced or metastatic disease, as well as those with resected NSCLC after radical surgery [98,99,100,101,102,103,104]. The ctDNA clearance after neoadjuvant treatment was associated with a significantly longer OS (HR 0.26, 95% CI 0.07–0.93) and PFS (HR 0.04, 95% CI 0.00–0.55) in patients enrolled in the NADIM II trial [105]. Results were similar in an exploratory analysis from the CheckMate 816 trial, where ctDNA clearance correlated with higher pCR rates (46% vs. 0% in patients without ctDNA clearance) and longer EFS (median NR vs. 18.9 months; HR 0.60, 95% CI 0.20–1.82) [48]. In addition, post-resection ctDNA positivity is not only prognostic but increases with disease stage and nodal status, as shown in an exploratory analysis from the IMpower010 trial [22]. Since these several studies individualized the rapid decay of ctDNA levels after surgery and the ctDNA detection after R0 resection as poor prognostic factors, being related with a decreased EFS, consequently, MRD could be used as surveillance and/or treatment decisions “biomarker” in this setting [98,99,106]. MERMAID-2 is a phase III trial that aims to determine the efficacy of durvalumab in stage II-III NSCLC after curative intent, using ctDNA as a marker of MRD and, therefore, as an enrolment criterion for adjuvant treatment; however, enrolment was halted due to insufficient patient recruitment [107]. In conclusion, patients with detectable ctDNA at baseline or after treatment, as well as those who did not clear ctDNA post-treatment, exhibited significantly worse clinical outcomes [108]. Consequently, ctDNA detection has the potential to identify patients who may benefit from further therapeutic intervention [109].

## 6. Surgical Outcome After Immunotherapy

The AEGEAN, CheckMate 816, IMpower010, KEYNOTE-091, Neotorch, and KEYNOTE-671 trials have confirmed the benefits of immunotherapy given as a neoadjuvant treatment in combination with chemotherapy [22,23,48,58,62,63]. Despite these findings, the risks of neoadjuvant treatment are not performing curative surgery, as well as delays in surgical resection, increased operative complexity, and morbidity or toxicities from induction therapy. The neoadjuvant trials comparing chemo-immunotherapy vs. chemotherapy alone reported similar or higher rates of surgery in patients who underwent combined chemo-immunotherapy, but 18% to 22% of patients did not undergo surgery due to disease progression, adverse events, and consent withdrawal by patients. The percentage of patients who did not undergo surgery because of disease progression was lower in the chemo-immunotherapy arm than in the chemotherapy arm. For instance, in the NEOTORCH trial, surgery was not performed in 17% vs. 26.7% of patients in the toripalimab and placebo arms, respectively; of these patients, 2.5% vs. 15.3% due to disease progression [63]. Similarly, in the NADIM II study, 13.7% of patients in the chemotherapy-only arm did not undergo surgery due to progression compared to 0% of patients in the chemo-immunotherapy arm [57]. The adjuvant treatment was administered to 73% vs. 69% of patients, with completion of the adjuvant treatment in 40% vs. 35% of them, in the KEYNOTE 671 trial, while in the NEOTORCH trial, 71% vs. 65% of patients received the treatment in the toripalimab vs. placebo arm, respectively; among them, 44% and 33% of patients completed 13 cycles of the adjuvant treatment [58,63]. Another major concern about chemo-immunotherapy is the potential for surgical delay. In the trials we reviewed, the percentage of patients with delayed surgery was comparable between chemo-immunotherapy and chemotherapy alone, with the majority of the delays lasting approximately two weeks for the majority of patients. For example, in the CHECKMATE 77T trial, surgery was delayed by less than 2 weeks for 55.6% of patients in the chemo-immunotherapy arm, and for 63.3% of patients in the chemotherapy-alone arm, 1.7 weeks (range: 0.6–3.0) and 1.1 weeks (range: 0.4–2.9). The median delay was a median of 1.7 weeks (0.6–3.0) in the chemo-immunotherapy arm and 1.1 weeks (0.4–2.9) in the chemotherapy-alone arm [61].

Data regarding the complexity of the procedure are elusive and not directly measured in most trials. Considering the type of surgery performed, pneumonectomies were generally less common in the chemo-immunotherapy group than in the chemotherapy-alone cohort (16.8% vs. 25.2% and 9% vs. 13.5%, in CheckMate 816 and CheckMate 77T, respectively) [48,61]. However, in KEYNOTE 671, the percentage of pneumonectomies was similar in the two arms (12%) [58]. Surgical complications had similar rates in patients treated with combined chemo-immunotherapy and those with chemotherapy alone, and the most frequently reported were pain, dyspnea, anemia, wound complications, and pneumonia. In the NEOTORCH trial, surgery-related postoperative adverse events were experienced by a least 10% of patients in either treatment group. In CheckMate 816, surgical complications occurred in 42% and 47% of patients in the chemo-immunotherapy group and chemotherapy-alone group, respectively, with similar rates of G ≥ 3 adverse events (12% and 15%) [48,63]. In the TD-FOREKNOW trial, among patients with surgical resection, surgery-related adverse events were reported for 16 of 40 patients (40.0%) in the camrelizumab + chemotherapy group and for 14 of 42 patients (33.3%) in the chemotherapy group, most of which were Grade 1 or 2 [50]. Perioperative mortality was similar between the two arms [58]. Achieving complete resection (R0) constitutes a key quality for evaluating the oncologic outcomes of surgery, leading to superior OS [19,26]. The R0 resection rate was higher in patients who underwent chemo-immunotherapy vs. chemotherapy alone in all trials, with percentages ranging from 83.3% to 100% [48].

Systematic lymph nodes sampling or dissection after neoadjuvant treatment is part of the standard radical surgery procedure and allows for better stratification of the disease stage and prognosis [110]. Some retrospective analyses suggest that the extent of lymphectomy may have an impact on ICIs efficacy, but evidence on this regard is, to date, insufficient to support modifying standard care [111,112].

## 7. Expert Opinion

The advent of immunotherapy for the treatment of resectable NSCLC in the neoadjuvant/perioperative and adjuvant setting has radically changed the approach for patients affected by resectable stage III disease. Indeed, the addition of immunotherapy to neoadjuvant chemotherapy in patients at stage III has shown to provide a reduction in relative risk of approximately 40%, with consistent data across different trials. It has also shown a higher rate of pCR compared to chemotherapy alone, with the pCR rate reaching, in many studies, a value ten times higher than that observed with the previous standard of care. Even in the adjuvant setting, immunotherapy has shown a clear improvement in outcomes, with a relative reduction in the risk of recurrence or death of over 20%. However, the most important advantages are currently limited to patients with high PD-L1 expression (>50%) if treated with adjuvant atezolizumab, while for therapy with pembrolizumab, the best subgroup of patients has not yet been identified, and the data relating to adjuvant durvalumab presented at ESMO 2024 were negative.

On the other hand, the complexity of treatment requires an organized multidisciplinary group. The speed of global and mediastinal staging through radiological, minimally invasive techniques (with EBUS currently preferred over mediastinoscopy), and the synergy between medical oncologists with thoracic surgeons and radiation oncologists is the key to the best outcomes. In this context, the expertise of the different specialists is essential to be able to guarantee the best therapeutic choices. For this reason, it is advisable to refer to lung units for the therapeutic decisions of a patient in a locally advanced stage. In this scenario of new potentially curative perioperative approaches, it is also to be considered that consensus on resectability of lung cancer is changing, and during 2023 WCLC, new perspectives about resectability were presented. In patients with N2 multistation, some T4 tumors, by infiltration of major structures and N2 bulky (only in highly selected cases), are to be considered potentially resectable, needing a multidisciplinary approach with case-by-case discussion, involving a range of specialists, including thoracic surgeons, radiation oncologists, oncologists or pulmonologists, pathologists, and imaging specialists [113,114]. Nowadays, molecular characterization plays a relevant role in therapeutic choices, even in the early setting of NSCLC. Indeed, we know how some gene alterations (i.e., *EGFR*, *ALK*, *ROS1*, and *RET*) can reduce the probability of response to immunotherapy regardless of PD-L1 expression. In some cases, neoadjuvant treatments must be excluded in favor of more effective adjuvant treatments, as in the case of *EGFR*-mutant NSCLC. For such patients, chemo-immunotherapy is not only ineffective but may also preclude adjuvant treatments, which have demonstrated very high efficacy in terms of DFS. Additionally, the cancer center expertise and the cohesion and synergy of the multidisciplinary team also play a critical role in the neoadjuvant treatment. In the case of adverse events or worsening of general conditions related to oncological treatment, they must be prepared to reassess staging and expedite surgery if needed. This approach helps prevent adverse events from compromising the curative potential of surgery. Finally, setting the correct patient pathway right from the diagnostic and staging phase is crucial. Based on current evidence, it must be considered wrong to start neoadjuvant treatment without having the certainty of operability. Despite the great downstaging capacity of these new treatments, the therapeutic strategy cannot be set on the probability of downstaging and tumor shrinkage. The risk, in such cases, is to deny the patient the best appropriate treatment represented by the combination of chemotherapy and radiotherapy, followed by immunotherapy or targeted therapy (e.g., for patients with an *EGFR* mutation). In this regard, the potential of evaluation of ctDNA before and after surgery has shown promise in predicting the patient’s outcome, risk of recurrence, and probability of responding to therapies. However, identifying more sensible and specific biomarkers would be a significant step forward, enabling the development of more personalized treatment strategies tailored to each patient.

## 8. Conclusions

Early-stage NSCLC management is an open question due to evolving criteria of resectability and the introduction of the new perioperative therapeutic approaches. The choice between an upfront surgery strategy with the option of an adjuvant therapy, rather than a neoadjuvant treatment, is still unanswered, highlighting the need for ad hoc prospective phase III trials to provide definitive indications. Additionally, it is essential to evaluate the incremental benefit of extending immunotherapy for an additional year after neoadjuvant chemo-immunotherapy and to assess whether an entire year of adjuvant immunotherapy is truly necessary to achieve meaningful clinical benefits. The negativity of ctDNA at the end of neoadjuvant therapy and before surgery predicts the response and defines the prognosis of the patient after surgery with extremely high accuracy. However, unlike the metastatic stage, detecting ctDNA in the early stage requires much more sensitive technologies and advanced expertise of molecular biologists in interpreting the data.

In conclusion, the early and locally advanced stages of NSCLC are being enriched with multiple therapeutic strategies that considerably increase the chances of actually curing patients affected by NSCLC. However, the complexity of this clinical condition requires great experience and attention from the staging and diagnostic point of view and requires multidisciplinary management. 

## Figures and Tables

**Table 1:** Principal phase II–III trials evaluating adjuvant treatment with immune checkpoint inhibitors in patients with resected non-small-cell lung cancer.

Study ID	Enrolment	Year	N° Pts	Regimens (Therapy; Maintenance)	Months of Adj	Stage	Excluded Drivers	EGFR/ALKm EXP vs. CRTL, %	Discontinuation for IO Toxicities (EXP vs. CTRL)	Median FUP, Months	HR EFS	G ≥ 3 Toxicities
Impower 010	Oct 2015–Sept 2019	2023	1280	Chemo + Atezo (1200 mg q21)/Chemo + BSC	12	IB–IIIA	NO	7.8 vs. 9.6	18 vs. 0	60	0.85	22 vs. 12
KN 091	Jan 2016–May 2020	2022	1177	Pembro (200 mg q21)/Placebo	12	IB–IIIA	NO	8 vs. 7	20 vs. 6	51.7	0.81	34.1 vs. 25.8
BR.31	NA	2024	1415	Durva (20 mg/kg q28)/Placebo	12	IB–IIIA	NO	NA	14 vs. 5.1	60	0.89	27 vs. 21

Legend: N°, numbers; pts, patients; adj, adjuvant; EXP, experimental; CRTL, control; FUP, follow-up; HR, hazard ratio; EFS, event-free survival; G, grade; Oct, October; Sept, September; Chemo, chemotherapy; Atezo, atezolizumab; BSC, best supportive care; NA, not applicable; Jan, January; Pembro, pembrolizumab; Durva, durvalumab.

**Table 2:** Principal phase II–III trials evaluating adjuvant treatment with tyrosine-kinase inhibitors in patients with resected non-small-cell lung cancer.

Study ID	Trial Phase	Year	N° Pts	EGFRm	Ex19del (EXP vs. CTRL)	L858R (EXP vs. CTRL)	Excluded Drivers	Regimens (Therapy; Maintenance)	Months of Adj	Stage	IA	IB	II	IIIA	Adjuvant CT, %	Median FUP, Months	DFS Rate	HR DFS	HR OS	G ≥ 3 Toxicities, %
BR19	III	2013	503	2.9	NA	NA	NA	gefitinib/placebo	24	IB–IIIA	NA	53 vs. 50	35 vs. 35	12 vs. 14	17 vs. 17	56	NA	1.22	1.24	41 vs. 16
ADJUVANT/ CTONG1104	III	2018	222	100	58 vs. 57	48 vs. 48	non-EGFR mutation Ex19del or L858R	gefitinib/adjuvant CT	24	II–IIIA	NA	NA	34 vs. 34	64 vs. 65	NA	80	5Y DFSR: 23.2 vs. 22.6	0.56	0.92	12 vs. 48
IMPACT	III	2021	232	100	64 vs. 59	52 vs. 56	non-EGFR mutation Ex19del or L858R	gefitinib/adjuvant CT	24	II–IIIB	NA	NA	36.2 vs. 35.4	63.8 vs. 64.6	NA	70	5Y DFSR: 31.8 vs. 34.1	0.92	1.03	41.7 vs. 92.1
SELECT	II	2018	100	100	62	35	non-EGFR mutation	erlotinib/placebo	24	IA–IIIA	14	31	27	28	NA	64	5Y DFSR: 56	NA	NA	22
RADIANT	III	2015	973	16.4	NA	NA	NA	erlotinib/placebo	24	IB–IIIA	NA	45.6	29.2	22.4	49.1	47	2Y DFS: 75 vs. 54	0.61	NR	43.1 vs. 15.5
CORIN	II	2023	128	100	50.8 vs. 46.2	46 vs. 52.3	non-EGFR mutation	icotinib/ placebo	12	IB	NA	100	NA	NA	0	39.9	3Y DFS: 96.1 vs. 84	0.23	NR	6.3
EVIDENCE	III	2021	322	100	53 vs. 53	47 vs. 47	non-EGFR mutation Ex19del or L858R	icotinib/adjuvant CT	2	IIA–IIIA	NA	NA	34 vs. 38	66 vs. 61	NA	24.9	3Y DFR: 63.9 vs. 32.5	0.55	0.91	11 vs. 61
ADAURA	III	2020	682	100	55 vs. 55	45 vs. 45	non-EGFR mutation Ex19del or L858R	adjuvant chemotherapy; osimertinib/placebo	36	IB–IIIA	NA	32 vs. 32	43 vs. 34	35 vs. 35	60 vs. 60	44.2	4Y DFS: 70 vs. 38	0.27	0.49	23 vs. 13
ALINA	III	2024	257	NA	NA	NA	NA	alectinib/chemotherapy	24	IB–IIIA	NA	10.8 vs. 9.4	34.6 vs. 33.9	53.1 vs. 55.1	NA	27.8	3Y DFR: 88.7 vs. 54	0.24	NA	29.7 vs. 30.8

Legend: N°, numbers; pts, patients; EGFRm, EGFR mutated; EXP, experimental; CRTL, control; adj, adjuvant; CT, chemotherapy; FUP, follow-up; DFS, disease-free survival; HR, hazard ratio; OS, overall survival; G, grade; NA, not applicable; Y, years; DFRS, disease-free survival rate; NR, not reached; DFR, disease-free relapse.

**Table 3:** Principal phase II–III trials evaluating neoadjuvant or perioperative treatment with immune checkpoint inhibitors in patients with resectable non-small-cell lung cancer.

Study ID	Enrollment	Trial Phase	Year	N° Pts	Regimens (Therapy; Maintenance)	Setting	Cycles of Neoadj IO	Months of Adj	Stage	Excluded Drivers	Surgery (EXP vs. CTRL), %	Adjuvant Therapy, %	Discontinuation for Toxicities (EXP vs. CTRL), %	Median FUP, Months	PCR, %	MPR, %	EFS Rate, %	HR EFS	HR OS	G ≥ 3 Toxicities, %
NADIM II	April 2017–August 2018	II	2022	86	CBDCA-PAC +/− Nivo (360 mg q21); Nivo (240 mg q14 for 4 mo, followed by 480 mg once q28 for 8 mo)	Perioperative	3	12	IIIA	EGFR; ALK	93.0 vs. 69	90.2	NA	26.1	36.8 vs. 6.9	52.6 vs. 13.8	2Y EFS 67.2 vs. 40.9	0.47	0.43	22 vs. 10
AEGEAN	January 2019–April 2022	III	2023	802	Chemo + Durva (1500 mg q21)/Placebo; Durva (1500 mg q28)/Placebo	Perioperative	3	12	IIA–IIIB	NA	80.6 vs. 80.7	81.4	12 vs. 6	11.7	17.2 vs. 4.3	33.3 vs. 12.3	2Y EFS: 63.3 vs. 52.4	0.68	NA	42.4 vs. 43.2
NEOTORCH	March 2023–June 2023	III	2023	501	Chemo + Tori (240 mg q 21)/Placebo; Tori (240 mg q 21)/Placebo	Perioperative	3	12	II–III	EGFR; ALK	82.2 vs. 73.3	71.3 vs. 64.9	9.4 vs. 7.4	18.3	24.8 vs. 1.0	48.5 vs. 8.4	2Y EFS: 64.7 vs. 38.7	0.40	0.62	63.4 vs. 54.0
KN 671	April 2018–Dec 2021	III	2023	797	Chemo + Pembro (200 mg q 21)/Placebo; Pembro (200 mg q21)/Placebo	Perioperative	4	12	II–IIIB	NA	82.1 vs. 79.5	73.2	12.6 vs. 5.3	25.2	18.1 vs. 4.0	30.2 vs. 11.0	2Y EFS: 62.4 vs. 40.6	0.59	0.72	45 vs. 38
CM 77T	Nov 2019–April 2022	III	2024	461	Chemo + Nivo (360 mg q 21)/Placebo; Nivo (480 mg q28)/Placebo	Perioperative	4	12	IIA–IIIB	EGFR; ALK	68.1 vs. 68.5	NA	28.6 vs. 17.0	25.4	25.3 vs. 4.7	35.4 vs. 12.1	1.5Y EFS 70.2 vs. 50.0	0.59	NA	32.5 vs. 25.2
RATIONALE 315	June 2020–August 2022	III	2024	453	Chemo + Tislelizumab (200 mg q21); Tislelizumab (400 mg q42)/Placebo	Perioperative	3 or 4	12	II–IIIA	EGFR; ALK	84.0 vs. 76.0	74.3 vs. 64.8	NA	22.0	41.0 vs. 6.0	56.0 vs. 15.0	2Y EFS: 68.0 vs. 52.0	0.56	0.62	72.0 vs. 66.0
CM816	March 2017–Nov 2019	III	2022	358	Chemo +/− Nivo (360 mg q21)	Neoadj	3	NA	IB–IIIA	EGFR; ALK	84.4 vs. 77.7	NA	15.9 vs. 15.4	57.6	24.0 vs. 2.2	36.9 vs. 8.9	4Y EFS: 49 vs. 38	0.66	0.71	33.5 vs. 36.9
TD-FOREKNOW	April 2020–January 2022	II	2023	94	Chemo + Camrelizumab (200 mg q 21)/Placebo	Neoadj	3	NA	IIIA–IIIB	EGFR; ALK	95.8 vs. 93.6	NA	2.12 vs. 2.12	14.1	32.6 vs. 8.9	65.1 vs. 15.6	2Y EFS 76.9 vs. 67.6	NA	NA	25.6 vs. 11.1
NeoCOAST	March 2019–September 2020	II	2023	83	Durva q28/Durva q28 + Ole q14/Durva q28 + Mona q14/Durva q28 + Durva q7	Neoadj	1	NA	IA3–IIIA	NA	81.0/90.0/93.8 vs. 84.6	NA	8.4	NA	3.7/9.5/10 vs. 12.5	11.1/19.0/30.0 vs. 31.3	NA	NA	NA	0/4.8/0 vs. 6.3
NEOSTAR	June 2017–Nov 2018	II	2021	44	Nivo (3 mg/kg q14)/Nivo (3 mg/kg q14) + Ipi (1 mg/kg q42)	Neoadj	3	NA	IB–IIIA	NA	NA	NA	NA	22.2	10 vs. 38	22 vs. 38	NA	NA	NA	13 vs. 10

Legend: N°, numbers; pts, patients; neoadj, neoadjuvant; IO, immunotherapy; EXP, experimental; CRTL, control; FUP, follow-up; PCR, pathological complete response; MPR, major pathological response; EFS, event-free survival; HR, hazard ratio; OS, overall survival; G, grade; CBDCA, carboplatin; PAC, paclitaxel; Nivo, nivolumab; NA, not applicable; Y, years; chemo, chemotherapy; Durva, durvalumab; Tori, toripalimab; Dec, December; Pembro, pembroliuzumab; Nov, November; Ole, oleclumab; Mona, monalizumab; Ipi, ipilimumab.
